# Case Report: Neo-ostium creation with saphenous vein in a patient with anomalous origin of the right coronary artery

**DOI:** 10.3389/fped.2023.1275345

**Published:** 2023-11-22

**Authors:** Bahar Temur, Zeynep Sıla Özcan, Serdar Başgöze, Selim Aydın, Füsun Güzelmeriç, Ersin Erek

**Affiliations:** ^1^ Department of Cardiovascular Surgery, Faculty of Medicine, Atakent Hospital, Acibadem Mehmet Ali Aydinlar University, Istanbul, Türkiye; ^2^ School of Medicine, Acibadem Mehmet Ali Aydinlar University, Istanbul, Türkiye; ^3^ Department of Pediatric Cardiovascular Surgery, Cengiz Gökçek Gynecology and Pediatrics Hospital, Gaziantep, Türkiye; ^4^ Department of Anesthesiology, Faculty of Medicine, Atakent Hospital, Acibadem Mehmet Ali Aydinlar University, Istanbul, Türkiye

**Keywords:** congenital heart disease, congenital heart surgery, coronary artery anomaly, coronary artery imaging, anomalous right coronary artery

## Abstract

Anomalous origin of the right coronary artery from the left sinus of Valsalva is a rare congenital anomaly. Most patients may be asymptomatic, but some may experience major cardiac events such as syncope, arrhythmias, and sudden cardiac death. We present a 16-year-old patient, who had several syncopes, with anomalous origin of the right coronary artery from the left coronary sinus, with an intramural and interarterial course between the pulmonary artery and the aorta. We describe a new surgical procedure of neo-ostium creation with a saphenous vein.

## Introduction

Anomalous origin of the right coronary artery (RCA) from the left sinus of Valsalva and having a course between the great vessels is a rare congenital anomaly, with a prevalence varying between 0.026% and 0.250% (1). The condition may remain asymptomatic in some patients, however, manifestations such as angina pectoris, myocardial infarction, heart failure, syncope, arrhythmias, and sudden cardiac death may be present (2). Patients with interarterial and/or intramural course and narrowed, slit-like coronary ostial orifice, carry a higher risk. Surgery is warranted in symptomatic patients. Usual techniques for repair are reimplantation and unroofing procedures. These techniques consist of creating a new RCA ostium (2, 3). Here, we report a new surgical procedure of neo-ostium creation with a saphenous vein in a patient who presented with a history of multiple syncopes.

## Case description

**Figure 1:**
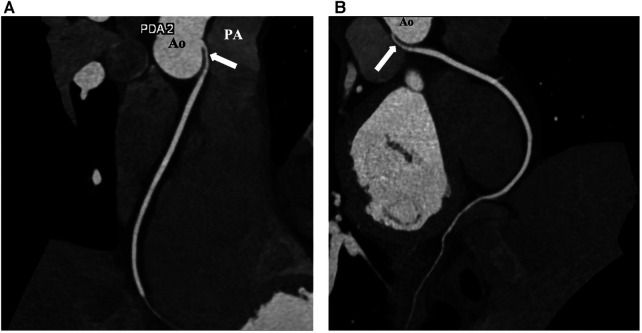
Preoperative coronary CT coronary images of the patient. (**A**) Image showing anomalous origin of the RCA ostium from the left coronary sinus and RCA (arrow) having an interarterial course between the aorta and pulmonary artery. (**B**) The stenotic intramural RCA segment (arrow). Ao, aorta; PA, pulmonary artery; RCA, right coronary artery.

**Figure 2:**
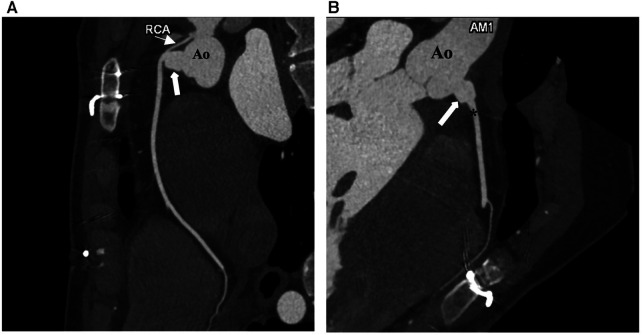
Postoperative CT coronary angiography images of the patient. (**A**) The proximal stenotic segment of the RCA (fine arrow) and the right coronary neo-ostium (bold arrow). (**B**) Saphenous vein graft (arrow) interposed between the RCA and the ascending aorta. Ao, aorta; RCA, right coronary artery.

**Figure 3:**
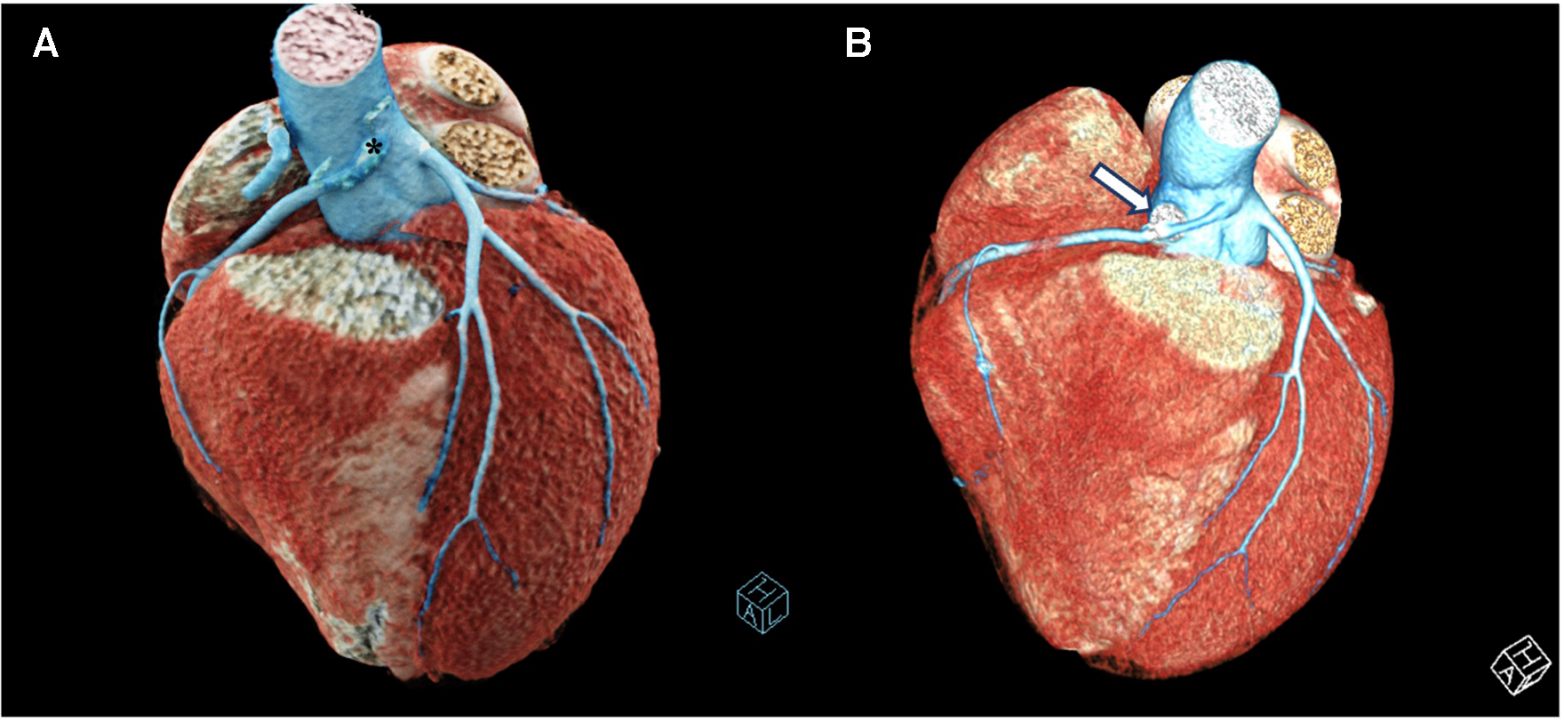
3d reconstructed CT images of the patient. (**A**) Preoperative 3D reconstructed CT image of the patient showing the anomalous origin of the RCA ostium (*) from the left coronary sinus. (**B**) Postoperative 3D reconstructed CT image of the patient showing the saphenous vein graft (arrow) interposed between the RCA and the ascending aorta creating a right coronary neo-ostium. RCA, right coronary artery.

The patient was a 16-year-old male adolescent, who had multiple syncopes during physical activity, the earliest one being 2 months before his admission. He underwent a thorough investigation to reveal the exact cause of his symptoms, including cardiologic, neurologic, hormonal, and psychiatric examinations. Nothing was found, except an anomalous origin of the RCA from the left coronary sinus, with an intramural and interarterial course between the pulmonary artery and the aorta. Electrocardiogram (ECG) and Holter monitoring showed no ischemic signs or arrhythmias. The echocardiographic examination was normal. Computerized tomography (CT) angiography confirmed the diagnosis with prominent ostial narrowing (Figures 1, 2A,B, 3A). Considering there was no other cause for the syncopes, surgery for anomalous RCA was planned.

The decision for surgical correction was made according to the ESC 2020 Guidelines for the Management of Adult Congenital Heart Disease (4). Although no ischemic/stress test was performed, the patient presented with unexplained syncopal events and high-risk anatomy, which constituted a class II indication for surgery in the guidelines.

The operation was performed with median sternotomy under general anesthesia. A short autogenous saphenous vein graft at the groin was harvested. Following the heparinization, aortic and right atrial cannulation were performed to initiate cardiopulmonary bypass (CPB). Diastolic cardiac arrest was achieved with antegrade tepid blood cardioplegia after cross-clamping at 32°C systemic hypothermia. After aortotomy, the right coronary ostium was identified originating from the left sinus of Valsalva and proximally coursing intramurally between the pulmonary artery and the aorta. A significant stenosis was visible at the RCA ostium. The RCA was prepared after the intramural course, near the anterior side of the aorta, where the original RCA ostium was supposed to be. An incision, 7–8 mm long, was made to the RCA, closer to the anterior side of the aorta. The saphenous vein graft was anastomosed to the arteriotomy end-to-side fashion using 7/0 propylene sutures. A small aortotomy was performed with a 4.5 mm aortic punch at the right sinus of Valsalva, at the closest point to the RCA arteriotomy. The rest of the saphenous vein was cut and anastomosed to the new aortic ostium. By this technique, a new RCA ostium was created with a very short saphenous vein (5–6 mm long) at the original position. Native RCA ostium and the intramural course were left intact. Aortotomy was closed. A cross-clamp was removed after de-airing. The patient was weaned from CPB uneventfully. CPB time and cross-clamp times were 66 and 39 min, respectively.

The patient was discharged uneventfully on the 6th day after surgery. Follow-up CT angiography 3 months after the operation demonstrated a wide patent neo-ostium of the RCA with an intact native course (Figures 2A,B; Figure 3B).

## Discussion

Anomalous origin of RCA from the left sinus of Valsalva is a rare congenital anomaly, which is usually considered innocuous. Follow-up is reasonable for asymptomatic patients, but it may be a cause of sudden death in the young (5). Although the exact mechanism of this phenomenon is not fully explained, some potential causes include ostial obstruction due to slit-like coronary orifice, compression of RCA between the aorta and pulmonary artery, stretching of the RCA with aortic/pulmonary artery distension, and the sharp angulation of the coronary artery with aortic/pulmonary artery distension (6). Angelini has shown variable lateral luminal compression of the intramural trunk that worsens during systole (7). The degree of lateral compression could be the reason for the different clinical outcomes observed in different patients.

In a study conducted by Lee et al. (8), the anomalous origin of the right coronary artery (RCA) from the left coronary sinus was classified into two high and low subtypes according to the location of the anomalous RCA ostium. The prevalence of typical angina and that of major adverse cardiac events were found to be significantly higher in those with an anomalous RCA with a high interarterial course, the ostium being located above the pulmonary valve. This results from the compression and stretching of the interarterial segment of the RCA between the great vessels during systole, when these vessels are distended (8, 9). Our patient had an anomalous RCA with a high interarterial and intramural course, thus being more at risk for major cardiac events.

Indications for surgical repair of anomalous RCA remain controversial and surgical correction is most frequently reserved for symptomatic patients and asymptomatic patients with high-risk morphologic abnormalities (10). Numerous surgical repair techniques have been reported in the literature for the repair of anomalous origin of the RCA. The optimal correction procedure is decided upon each patient's specific morphology. Coronary unroofing, ostioplasty, coronary reimplantation, translocation of the pulmonary artery (PA), and coronary artery bypass grafting using an internal thoracic artery or saphenous vein graft are techniques that are used for the correction of this anomaly (7, 10). Stent implantation may be another choice but after this procedure, chest pain may remain because of the intramural path (11). In patients with a single coronary ostium and no intramural course, coronary artery bypass grafting may be an option but this technique presents concerns about competitive flow (12). As an alternative, PA translocation is another option in pathologies without an intramural course. Unroofing is a suitable technique, especially in infants but with potential risks as the smaller size of the involved artery and its thinner wall may increase the risk of bleeding, future scarring, and occlusion (13). Besides, close proximity to the aortic valve commissure may necessitate commissural take-down procedure during unroofing, which may cause aortic valve insufficiency later on. Unroofing techniques may cause atherosclerosis and scarring in the long term due to significant manipulation of the coronary artery (14). In a study conducted by Arcieri et al. it was suggested that coronary unroofing is not a technique suitable for all anatomic subtypes of anomalous origin of coronary arteries and that the selection of a surgical technique must be done according to a specific patient anatomy-based approach (15).

In our case, the RCA had an interarterial and intramural course with a stenotic ostium. The aortic commissure was closer to the RCA ostium, which could make the unroofing procedure more complicated. Our new technique of neo-ostium creation is easier to perform with safe results. Leaving the native coronary arterial course intact is an advantage, in case saphenous vein dysfunction occurs. The same neo-ostial creation may also be performed with the autologous pericardium, but we preferred a short saphenous vein interposition because the thin saphenous vein wall is very suitable for coronary anastomosis and has demonstrated good long-term results. Although it seems that the right coronary ostium appears a little dilated in the control CT angiography, it actually reflects the natural diameter of the saphenous vein graft. We preferred to harvest the saphenous vein from the groin because it is larger in diameter and stronger in this area than the ankle region. In addition, the long-term graft durability is better and the stenosis risk is lower. Future aneurysmatic dilatation may be a concern, but it is rare, as far as we know from the very large data about the fate of saphenous vein grafts in coronary artery bypass operations. Increasing aneurysm size may be associated not only with a higher risk of rupture but also with high morbidity and mortality (16). Our patient will use acetylsalicylic acid (ASA) lifelong to prevent any complications. As an anomalous right coronary artery arising from the left coronary cusp with an interarterial course between the pulmonary artery and aorta is considered a high-risk subtype and sudden cardiac death during rest or sleep can be encountered in these cases, a specific surgical approach considering all the aspects of the patient anatomy should be employed to treat these patients (17).

Our new technique may be considered a bypass grafting type of procedure by some authors. However, these two techniques should be distinguished firstly due to, the saphenous vein, which we used, being very short (5–6 mm long) as a new ostium so that the atherosclerotic changes would be less than it would be with standard long vein bypass grafts. A new ostium can be created with minimal coronary manipulation with our new technique, which might be another advantage. Competitive flow from the native ostium will be negligible due to the short and large opening and may be protective in case of failure of the anastomosis. This technique also has a lower risk of proximal coronary artery stenosis or kinking compared with coronary artery translocation. The postoperative CT images (Figures 2A,B) presented in the study are from the third-month follow-up of our patient. The patient has no residual signs or symptoms and is in an overall good condition.

In conclusion, in patients with an anomalous right coronary artery from the left aortic sinus, with an interarterial and intramural course and a stenotic orifice, the creation of a neo-ostium using an autologous saphenous vein graft at the correct anatomic position may represent a sufficient repair strategy that effectively addresses this anomaly. Our technique might be a safe, easy, and reproducible alternative repair approach.

## Data Availability

The original contributions presented in the study are included in the article/Supplementary Material, further inquiries can be directed to the corresponding author.
